# Glucagon Increases Beating Rate but Not Contractility in Rat Right Atrium. Comparison with Isoproterenol

**DOI:** 10.1371/journal.pone.0132884

**Published:** 2015-07-29

**Authors:** Beatriz Merino, Ivan Quesada, Jesús Hernández-Cascales

**Affiliations:** 1 Instituto de Bioingeniería, Universidad Miguel Hernández, Elche, Spain; 2 CIBER de Diabetes y Enfermedades Metabólicas Asociadas (CIBERDEM), Elche, Spain; 3 Departamento Farmacología; Facultad de Medicina; Universidad Murcia, Murcia, Spain; Cinvestav-IPN, MEXICO

## Abstract

This study evaluated the chronotropic and inotropic responses to glucagon in spontaneously beating isolated right atria of rat heart. For comparison, we also investigated the effects resulting from stimulating β-adrenoceptors with isoproterenol in this tissue. Isoproterenol increased both atrial frequency and contractility but glucagon only enhanced atrial rate. The transcript levels of glucagon receptors were about three times higher in sinoatrial node than in the atrial myocardium. Chronotropic responses to glucagon and isoproterenol were blunted by the funny current (I*f*) inhibitor ZD 7288. Inhibitors of protein kinase A, H-89 and KT-5720 reduced the chronotropic response to glucagon but not to isoproterenol. Inhibition of ryanodine receptors and calcium/calmodulin dependent protein kinase II (important regulators of sarcoplasmic reticulum Ca2^+^ release), with ruthenium red and KN-62 respectively, failed to alter chronotropic responses of either glucagon or isoproterenol. Non selective inhibition of phosphodiesterase (PDE) with 3-isobutylmethylxantine or selective inhibition of PDE3 or PDE4 with cilostamide or rolipram respectively did not affect chronotropic effects of glucagon or isoproterenol. Our results indicate that glucagon increases beating rate but not contractility in rat right atria which could be a consequence of lower levels of glucagon receptors in atrial myocardium than in sinoatrial node. Chronotropic responses to glucagon or isoproterenol are mediated by I*f* current but not by sarcoplasmic reticulum Ca2^+^ release, neither are regulated by PDE activity.

## Introduction

Glucagon is a polypeptide hormone produced and secreted by the alpha cells of the pancreatic islets of Langerhans which is considered to induce cardiostimulatory effects [1]. Consequently, it is used for the treatment of poisoning caused by cardiodepressant drugs such as β-adrenoceptors blockers or calcium channel blockers [2]. The positive inotropic effect of glucagon has been established in ventricular myocardium [3–5], but their atrial effects are less well know. Indeed, glucagon seems to be devoid of inotropic effect [5,6] but it produces a positive chronotropic response in atrial myocardium [7]. The reason behind the differences observed between the atrial inotropic and chronotropic effects of glucagon is unknown but it may result from a different levels of glucagon receptor in atrial myocardium and sino-atrial node. Indeed, regional differences in density of glucagon receptors, have been detected in the heart [5] but whether or not their density in sinoatrial node is different than in the rest of the atrial myocardium has not been determined yet.

Cardiac effects of glucagon are due to stimulation of glucagon receptors associated with Gs protein, which causes adenylyl cyclase activation and the consequent increase of 3’,5’-cyclic adenosine monophosphate (cAMP) production [1]. In fact, mechanical actions of glucagon, in the heart, are cAMP/Protein kinase A (PKA) dependent [8] and they are limited by the activity of the enzymes cyclic nucleotide phosphodiesterases (PDEs) which provide the only mechanism for degrading cAMP [9]. The mechanism responsible for the chronotropic effect of glucagon is less well known. The sinoatrial node (located in a very small discrete area of the right atria near where the superior vena cava enters this chamber), is the primary pacemaker of the heart, and the determinant of cardiac automaticity and generation of the heart beat [10]. The “funny current” (I*f*), an inward current carried by Na^+^ and K^+^ ions, which is specifically activated at hyperpolarized membrane potentials, has been considered the most important determinant of cardiac automaticity [11]. Hyperpolarization-activated cyclic nucleotide gated channels (HCN) are the molecular correlate of I*f* and they are regulated by cAMP which facilitates their activation when binding to the channel [12]. More recently, spontaneous, rhythmic sarcoplasmic reticulum Ca^2+^ release, via ryanodine receptors (RyRs), has also been implicated as a vital factor in the generation of sinoatrial node spontaneous firing by activating an inward Na^+^-Ca^2+^ exchange current which accelerates the pacemaker firing [13]. This process requires basal phosphorilation of RyRs by both PKA and calcium/calmodulin dependent protein kinase II (CaMKII) [13]. cAMP activate PKA as well as CaMKII, the later by the cAMP target EPAC (exchange protein directly activated by cAMP), which traduce its effects via protein kinase C (PKC)/CaMKII [14]. PDE activity (preferentially PDE3 and PDE4 subtypes), by regulating basal cAMP levels, potently controls sinoatrial node rate as evidenced by the fact that suppression of PDE activity led to a ~ 55% increase in the spontaneous sinoatrial node cells beating rate [15].

Although it is known that the chronotropic effect of glucagon is cAMP related [1, 3], the possible involvement of the above mentioned mechanisms in this effect is unknown. The purpose of the present work was to study the responses of the right atria from the rat heart to glucagon. For comparison, we have also studied, in the same tissue, the effect of the activation of β-adrenoceptors by isoproterenol, which also produces a cAMP dependent positive inotropic and chronotropic effects by activating Gs/adenylyl cyclase/cAMP/PKA pathway [16]. We also evaluated glucagon receptors distribution in sinoatrial node and atrial myocardium as well as the possible involvement of the above mentioned mechanisms in the effect of glucagon in these tissues. In this study, we observed an increased presence of glucagon receptor transcript in the sinoatrial node compared with the atrial tissue.

## Methods

The study was performed in accordance with the European Union Council Directive of 22 September 2010 (2010/63/EU) and reviewed and approved by the Ethical Committee of the University of Murcia.

Male Sprague-Dawley rats weighting 250–350 g were kept under standardized conditions: 12 h-light/dark circle, 22°C and 70% humidity. Food and water were available *ad libitum*. Rats were rendered unconscious immediately upon cerebral concussion (percussive blow to the head) and euthanized by rapid exsanguination, after which the chest was opened and the heart rapidly removed and placed in Tyrode solution of the following composition (mmol/L): NaCl 136.9, KCl 5.0, CaCl_2_ 1.8, MgCl_2_ 1.5, NaH_2_PO_4_ 0.4, NaHCO_3_ 11.9 and Dextrose 5.0.

### Real-time PCR

The spontaneously beating right atria was carefully dissected. The sinoatrial node area, located in the upper left section of the right atria near where the superior vena cava enters this chamber [17], was excised from the rest of the atria under magnifying glasses aided view. Its identity was confirmed by the observation that sinus rhythm was maintained in the atrial section containing sinoatrial node but it disappears in the rest of the atrial myocardium. Quantitative PCR assays were performed using CFX96 Real Time System (Bio-Rad, Hercules, CA) and 7500 Real Time PCR System (Applied Biosystems, Foster City, CA). Total mRNAs were extracted from heart tissue using TriPure Isolation Reagent (Roche), and 1 μg of RNA was used for retrotranscription reaction (HighCapacity cDNA Reverse transcription, Applied Biosystems). Reactions were carried out in a final volume of 10 μl, containing 200 nM of each primer, 1 μl of cDNA, and 1×IQ SYBR Green Supermix (Bio-Rad). Samples were subjected to the following conditions: 10 min at 95°C, 40 cycles (10 s at 95°C, 7 s at 60°C, and 12 s at 72°C), and a melting curve of 63–95°C with a slope of 0.1 C/s. The reference gene was ribosomal protein large P0, also known as 36B4, and it was used as the endogenous control for quantification. The resulting values were analyzed with CFX Manager Version 1.6 (Bio-Rad), and values were expressed as the relative levels respect to control levels (2−ΔΔCT). Specific primers used for gene levels analysis as well as accession numbers and amplicon length are shown in S1 Table.

### Recording of right atrial contractility

The right atria was carefully dissected and mounted vertically in a 30-ml double walled glass chamber filled with Tyrode solution, gassed continuously with 95% O_2_-5% CO_2_ and maintained at pH 7.4 and 36°C. The lower end of the right atrium was fixed on a hook and the upper end was connected by a silk thread to an isometric force-displacement transducer (Grass FT-03). A preload tension of 0.5 g was applied to the atria and the tissues were allowed to equilibrate for 1 hour. Contractions were recorded and displayed on a computer screen using a Stemtech amplifier (Stemtech Inc., Houston, Texas) and ACODAS software (Dataq Instruments, Inc., Akron, Ohio). Tissues were allowed to equilibrate for 45–60 min before drug challenge.

#### Experimental protocols

Cumulative concentration-response curves to glucagon and isoproterenol were determined by increasing, stepwise, the concentration of each agent by 0.5 log unit. A maximal effect was achieved within 5 min after each concentration. Concentration-response curves for each agent were also performed after 30 minutes in the presence of either ZD 7288 (0.5 μmol/L) or ruthenium red (10 μmol/L) which effectively inhibit I*f* [18] and sarcoplasmic reticulum Ca^2+^ release [19], respectively. When examining the influence of the enzymes PKA, PKC, CaMKII or PDEs on the chronotropic effects of glucagon and isoproterenol, the concentration-response curves, for these two agents, were performed 30 minutes after the application of inhibitors of the mentioned enzymes. The inhibitors used were KT-5720 and H-89 for PKA [20,21], calphostin for PKC [22], KN-62 for CaMKII [23], and 3-isobutylmethylxantine (IBMX), cilostamide and rolipram which are respectively non selective PDE inhibitor and selective PDE3 and PDE4 inhibitors [9]. Drugs were added to the organ bath in a volume smaller than or equal to 0.1 ml. Only one concentration-response curve for glucagon or isoproterenol either alone or in the presence of each of the above indicated inhibitors was determined in the same atrial tissue. Control values refer to the frequency before the addition of drugs and the effect of a given drug was considered the change in control frequency after drug addition. Because some of the above agents (such as inhibitors of PDE or I*f*), changed the spontaneous beating rate, the beating rate in the presence of each compound was taken as the basal beating rate. The frequency was expressed as beats min^–1^ and contractility in mN.

### Drugs

Glucagon was generously supplied by Novo Nordisk Pharma S.A. (Madrid, Spain). Isoproterenol, ZD7288, ruthenium red, H-89, KT-5720, KN-62, calphostin, IBMX, cilostamide and rolipram were obtained from Tocris Bioscience (Madrid, Spain) and dimethyl sulphoxide (DMSO) from Probus, (Barcelona, Spain).

Glucagon, KN-62, calphostin, KT-5720, rolipram, cilostamide and IBMX were dissolved in DMSO and Tyrode solution (20% DMSO in Tyrode) and isoproterenol, H-89, ZD7288 and ruthenium were dissolved in Tyrode solution. This stock solution was diluted into pre-warmed and pre-aerated bathing solution to achieve the final concentration desired. The drug was added to the organ bath at an appropriate concentration so that the concentration of DMSO in the test solution was less than 0.3%, which produced no effect in these preparations.

### Statistical analysis

The results are expressed as mean values ± SE. Concentration-response curves were fitted with non-linear regression sigmoidal concentration-response curve, variable slope and–log EC_50_ and the maximal effect (E_max_) values were estimated from the concentration-response curves depicted using the GraphPad 5 Software Inc. San Diego, CA, USA).

Student’s t-test or one-way analysis of variance followed by the Newman Keuls post-hoc test for multiple comparisons were used. The criterion for significance was that P values should be less than 0.05.

## Results

### Effects of glucagon and isoproterenol

**Fig 1:**
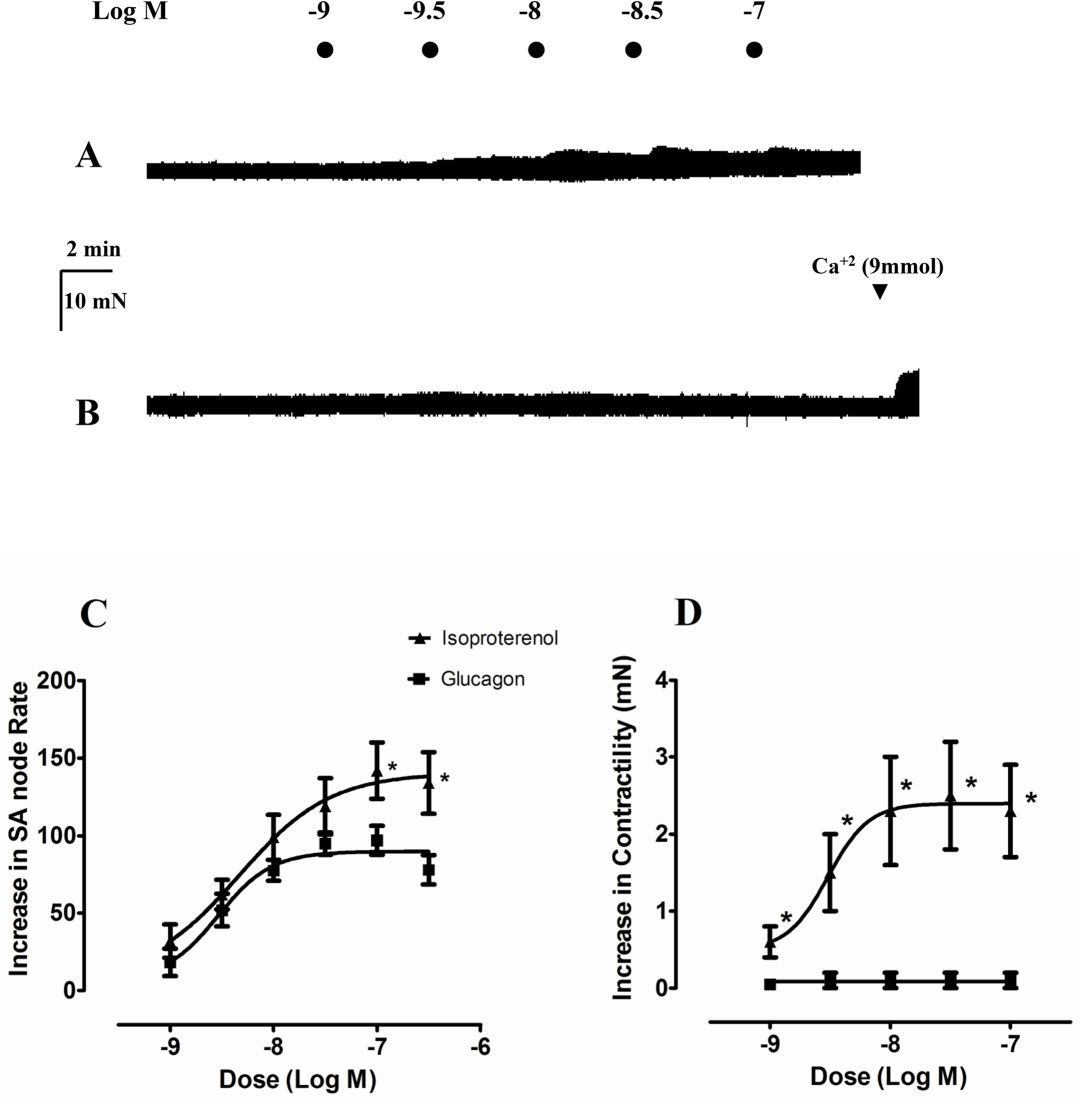
Effect of glucagon and isoproterenol on spontaneous beating rate and basal force of contraction in rat right atria. Representative traces showing that isoproterenol increases atrial contractility (A). In contrast, glucagon is virtually devoid of inotropic effect in a right atria which responds to Ca^2+^ (B). Cumulative concentration-response curves for the chronotropic (C) and inotropic (D) effects of glucagon (**■**) and isoproterenol (**▲**). Chronotropic and inotropic responses are expressed as increase in basal rate (278 ± 7 beats min^-1^) and contractility (3.3 ± 0.4 mN), respectively. Each point represents the mean value ± s.e.m. (vertical bars) of 5–12 experiments.

Typical responses of atrial contractility to glucagon and isoproterenol are illustrated in Fig 1A and 1B. As can be seen isoproterenol produced a concentration dependent positive inotropic and chronotropic effects (Fig 1C and 1D). However, glucagon produced chronotropic but not inotropic responses in this tissue (Fig 1C and 1D). The glucagon solvent DMSO, at the same concentrations, was devoid of effect in this preparation (data not shown).

### Glucagon receptor levels

**Fig 2:**
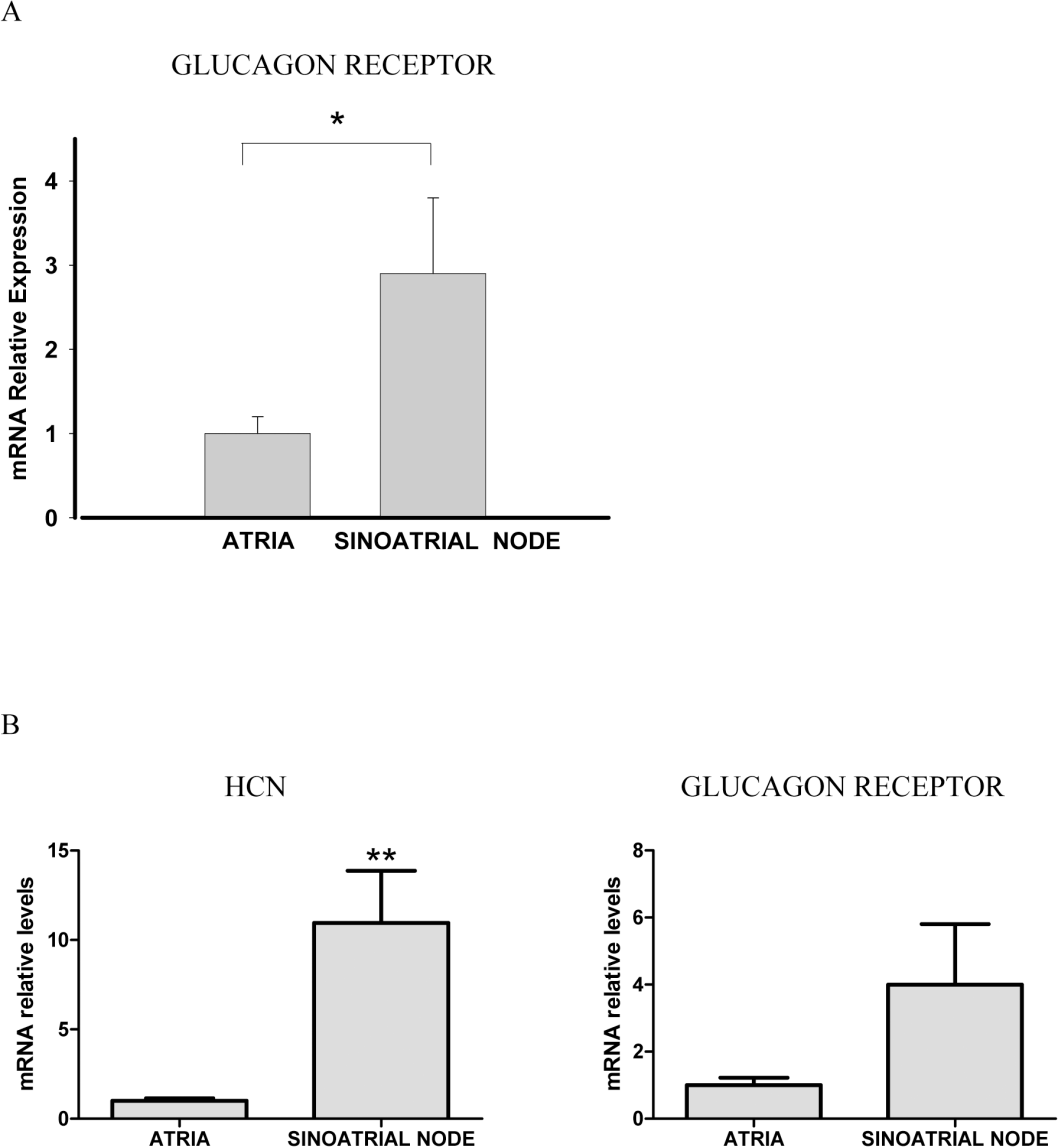
(A) The glucagon receptor mRNA levels are different in sinoatrial node and atrial myocardium. Relative quantification of glucagon receptor mRNA levels in sinoatrial node and myocardium of rat right atria. Values are expressed as mean ± s.e.m of 3–6 experiments. *P<0.05. (B) HCN and glucagon receptor mRNA levels in sinoatrial node and atrial myocardium. Values are expressed as mean ± s.e.m of 6–7 experiments. **P<0.01.

We analyzed the mRNA levels of the glucagon receptor comparing the heart sinoatrial node with the rest of the atria, which was used as control. Glucagon receptor mRNA levels was about three times higher in the sinoatrial node than in the atrial myocardium Fig 2A (p<0.05). The isolation and functional identity of the sinoatrial node area was performed as described in the methods section. In addition to the functional identification, in a different analysis we aimed to identify the sinoatrial node by the abundance of HCN levels in the samples. It has been reported that HCN channels are profuse in the region of the sinoatrial node but absent in the rest of the atrial region [10]. HCN levels was about 10 times higher in the sinoatrial node compared with the atria (Fig 2B). In line with the previous results, we observed that glucagon receptor levels was about 4 times higher in the sinoatrial node but, due to data dispersion, these values were not statistically significant (p = 0.1). In any case, because of the dissection protocol to extract the sinoatrial node, it may also contain surrounding tissue (atria), which may decrease the density of the glucagon receptor mRNA levels. Thus, glucagon receptor levels might be even higher in the sinoatrial node than the values shown here.

### Effects of inhibition o If and RyRs

**Fig 3:**
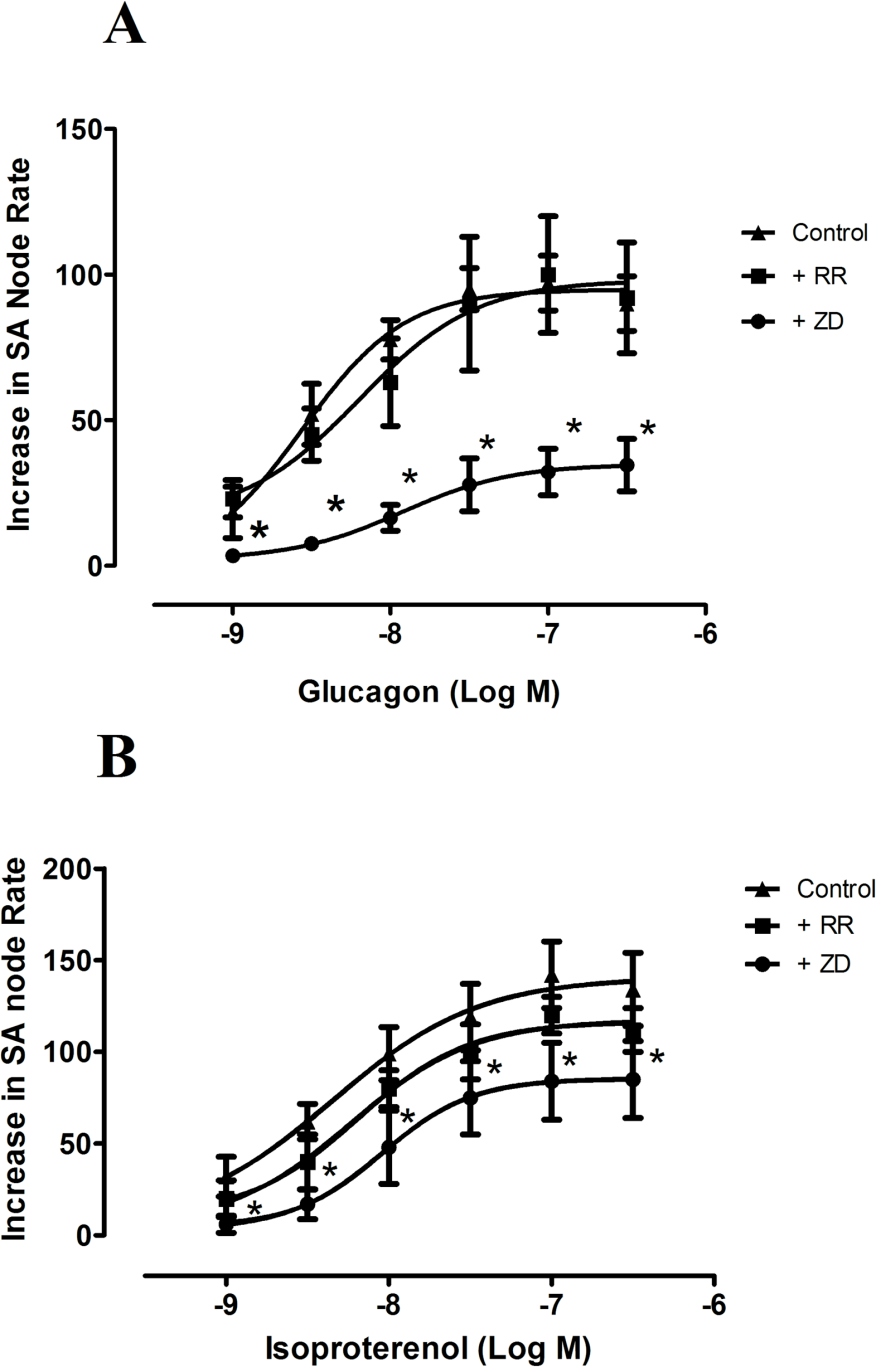
Concentration response curves for the positive chronotropic effects of glucagon (A) and isoproterenol (B), in the absence (▲) and in the presence of ruthenium red (10 μmol/L, ■), or ZD 7288 (0.5 μmol/L, ●) in the spontaneously beating rat right atria. Results are expressed as increase in basal rate (beats min^-1^). Ruthenium red and ZD 7288 reduced basal rate by 23 ± 3 beats min^-1^ (n = 7) and 93 ± 12 beats min^-1^ (n = 11), respectively. When the preparation was treated with either ruthenium red or ZD 7288, the beating rate in the presence of each of these agents was taken as the basal beating rate. Each point represents the mean value ± s.e.m. (vertical bars) of 4–6 experiments.

To investigate the role of I*f* in the positive chronotropic effect of glucagon, we performed concentration response curves for glucagon in the absence and in the presence of the I*f* inhibitor ZD 7228. The application of 0.5 μmol/L ZD 7228 to the organ bath, containing the right atria, reduced atrial rate by 93 ± 12 beats min^-1^ (n = 5), from the basal rate of 278 ± 7 beats min^-1^ (n = 12, P<0.05) and also reduced the chronotropic effects of glucagon and isoproterenol (Fig 3A and 3B, Table 1). We also studied whether sarcoplasmic reticulum Ca^2+^ release through RyRs was involved in the chronotropic responses to glucagon and isoproterenol. To this purpose, we investigated the chronotropic effect of these two agents in the absence and in the presence of the RyRs inhibitor ruthenium red (10 μmol/L). As can be seen in Fig 3A and 3B, ruthenium red which on its own reduced sinoatrial frequency by 23 ± 3 beats min^-1^ (n = 7, P<0.05) failed to modify the chronotropic responses to glucagon or isoproterenol in the right atria (Fig 3, Table 1).

**Table 1:** Chronotropic E_max_ and-log EC_50_ values for glucagon and isoproterenol in rat right atria in the absence and presence of different agents.

		Glucagon			Isoproterenol	
Drugs	n	E_max_	-log EC_50_	n	E_max_	-log EC_50_
_	6	97±9.2	8.7±0.10	6	142±18	8.6±0.14
*ZD 7288*	*5*	*34*.*6*±*9* *	*7*.*8*±*0.35* *	*6*	*85*.*8*±*21* *	*8*.*0*±*0.10* *
Ruthenium red	4	100.6±20	8.4±0.27	4	117±10	8.4±0.12
KN-62	5	103.6±17	8.7±0.17	4	137±5.3	8.8±0.33
Calphostine	4	110±8.2	8.5±0.06	_	_	_
H-89	4	60.2±11.5*	7.8±0.12*	4	140±45.6	8.6±0.25
KT-5720	5	63.6±8.7*	7.8±0.2*	4	140±15	8.4±0.3
IBMX	4	78.5±7.7	8.4±0.24	4	129±19.9	8.7±0.13
Cilostamide	4	86±8.9	8.5±0.25	_	_	_
Rolipram	5	93.2±6.0	8.3±0.29	_	_	_

*P<0.05 when compared with either glucagon or isoproterenol alone

### Effect of inhibition of PKA and EPAC

**Fig 4:**
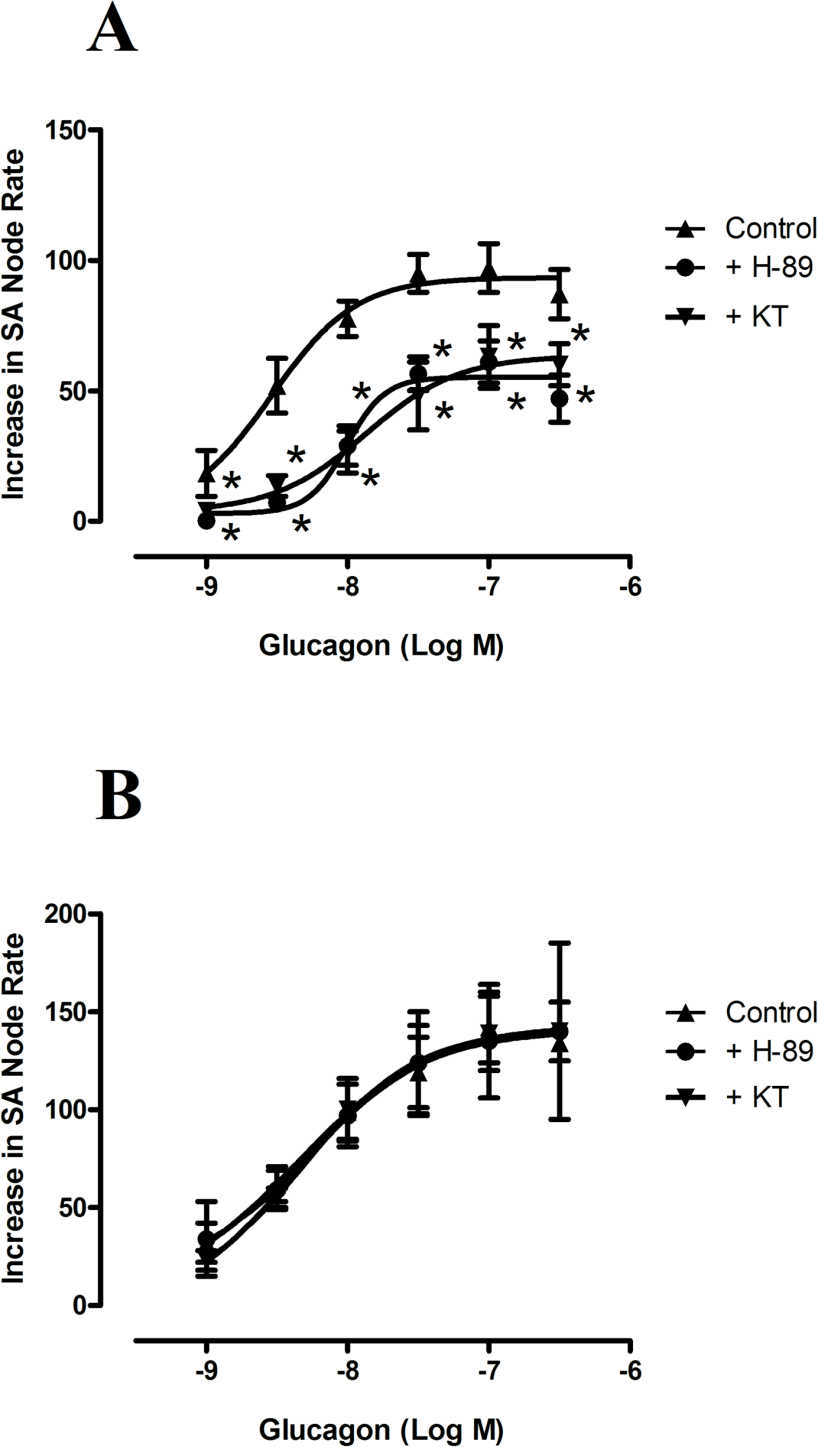
Concentration response curves for the positive chronotropic effects of glucagon (A) and isoproterenol (B), in the absence (▲) and in the presence of KT-5720 (0.5 μmol/L,▼), or H-89 (3 μmol/L, ●) in the spontaneously beating rat right atria. Results are expressed as increase in basal rate (beats min^-1^). When the preparation was treated with H-89, the beating rate was reduced by 46 ± 4 beats min^-1^ (n = 6), and was taken as the basal beating rate. Each point represents the mean value ± s.e.m. (vertical bars) of 4–6 experiments.

0.5 μmol/L10 of the PKA inhibitor KT-5720, which inhibits ~90% PKA activity [20], did not modify atrial frequency but reduced the positive chronotropic effect induced by glucagon (Fig 4A, Table 1). However, KT-5720 did not affect the chronotropic response to isoproterenol (Fig 4B, Table 1). Also, 3 μmol/L of H-89, which inhibits PKA activity by 96% [21], decreased atrial rate by 46 ± 4 beats min^-1^(n = 6, P<0.05) and further reduced the positive chronotropic effect of glucagon but still failed to modify the effect of isoproterenol on atrial rate (Table 1, Fig 4A and 4B). Therefore, these results indicate that PKA mediate the chronotropic response to glucagon but not to isoproterenol

**Fig 5:**
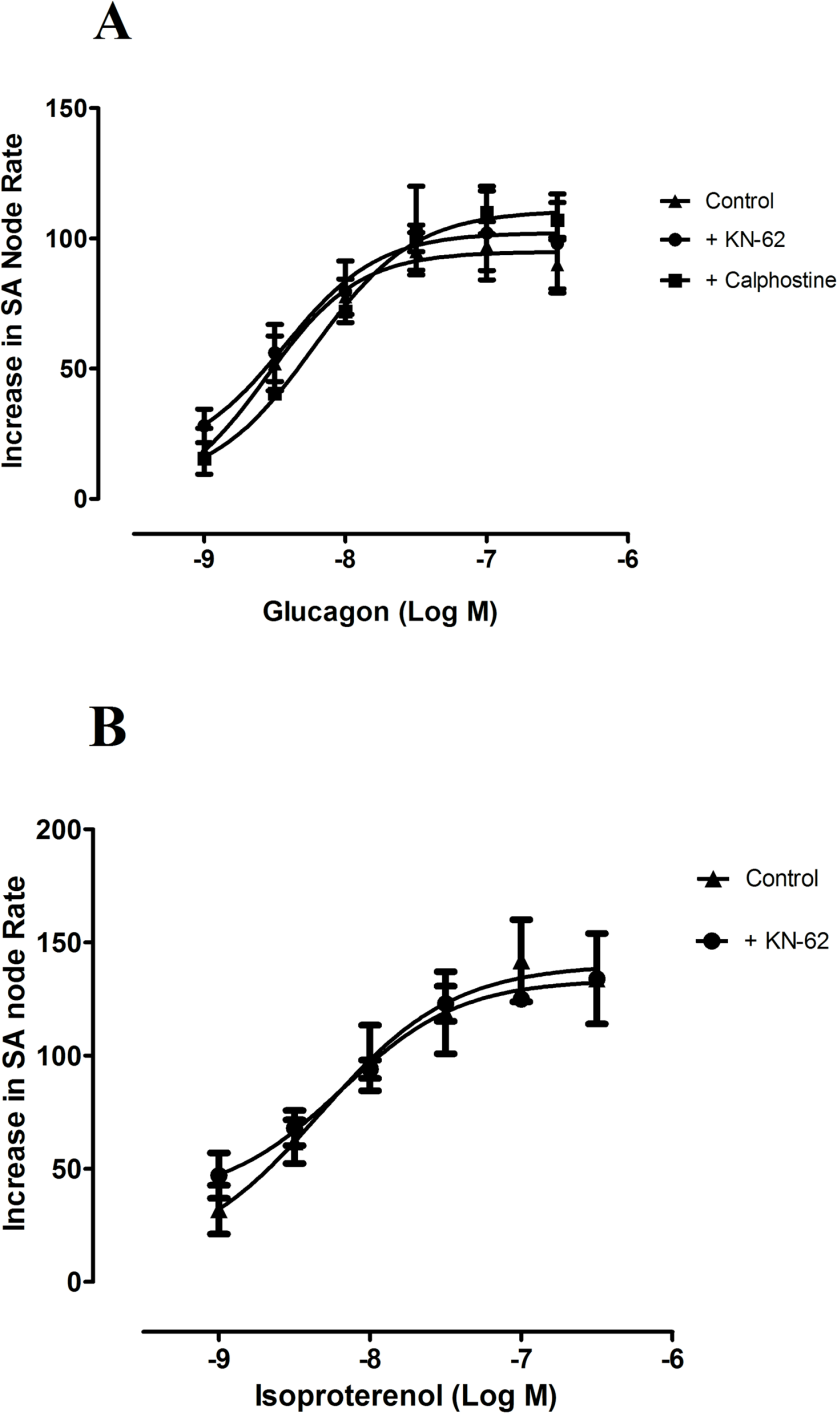
Concentration response curves for the positive chronotropic effects of glucagon (A) and isoproterenol (B), in the absence (▲) and in the presence of KN-62 (3 μmol/L,●), in the spontaneously beating rat right atria. Chronotropic effect of glucagon in the presence of calphostine (0.5 μmol/L, ■) was also studied. Results are expressed as increase in basal rate (beats min^-1^). When the preparation was treated with KN-62 the beating rate was reduced by 17 ± 7 beats min^-1^ (n = 8) and it was taken as the basal beating rate. Each point represents the mean value ± s.e.m. (vertical bars) of 4–6 experiments.

We also investigated the possible involvement of EPAC, which traduces its effects via PKC/CaMKII pathway [14], using inhibitors of either PKC and CaMKII. An inhibition of PKC activity of 90% with 0.5 μmol/L calphostin [22], tended to reduce atrial rate (-8 ± 3 beats min^-1^, n = 4, P>0.05) but did not alter the positive chronotropic effect of glucagon (Table 1. Fig 5A). Also, 1 μmol/L of KN-62, which inhibits CaMKII activity by ~50% [23], reduced atrial rate by 17 ± 7 beats min^-1^ (n = 8, P<0.05), but not modified the chronotropic effects of either glucagon or isoproterenol (Table 1, Fig 5A and 5B). From these results we conclude that EPAC pathway does not seem to mediate the chronotropic responses to either glucagon or isoproterenol. To further investigate the possible role of EPAC, we used the EPAC inhibitor ESI-09, but this agent decreased inotropy, abolished atrial rate and produced tetanic contraction of the tissues (S1 Fig), thus precluding the study of interactions between this drug and either glucagon or isoproterenol.

### Effect of PDEs inhibition

**Fig 6:**
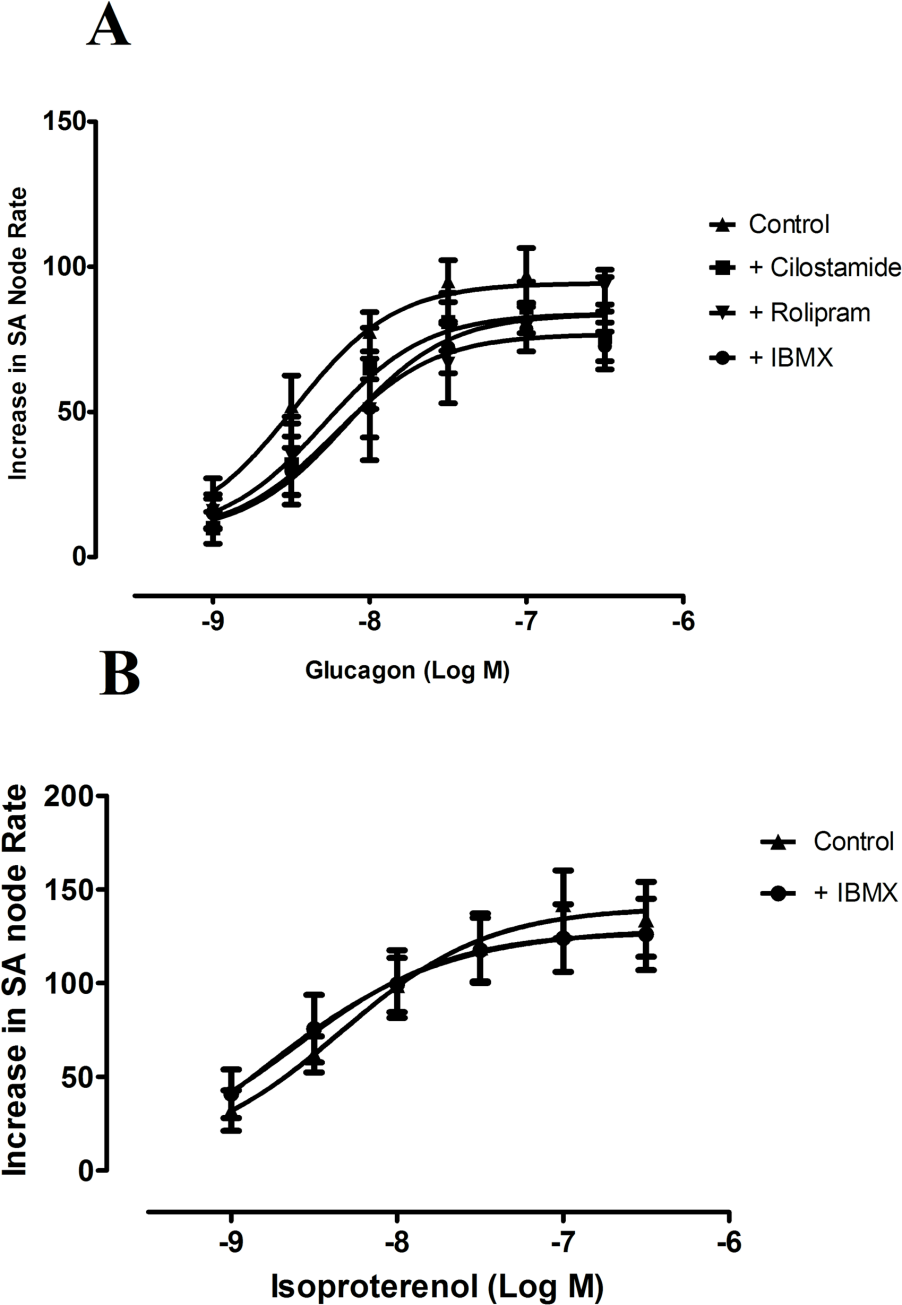
(A) Concentration response curves for the positive chronotropic effects of glucagon in the absence (▲) and in the presence of the non selective PDE inhibitor IBMX (3 μmol/L, ●), the selective PDE3 inhibitor cilostamide (0.3 μmol/L, ■) or the selective PDE4 inhibitor rolipram (1 μmol/L, ▼) in the spontaneously beating rat right atria. (B) Concentration response curves for the positive chronotropic effects of isoproterenol in the absence (▲) and in the presence of the non selective PDE inhibitor IBMX (3 μmol/L, ●) in the spontaneously beating rat right atria. Results are expressed as increase in basal rate (beats min^-1^). Both IBMX and rolipram increased beating rate by 26 ± 3 beats min^-1^ (n = 8) and 36 ± 6 beats min^-1^ (n = 5) respectively. Cilostamide did not changed basal atrial rate. When the preparation was treated with either IBMX or rolipram the beating rate in the presence of each of these agents was taken as the basal beating rate. Each point represents the mean value ± s.e.m (vertical bars) of 4–6 experiments.

In order to see whether PDEs inhibition modifies the effects of either glucagon or isoproterenol, on atrial rate, we tested the effects of these two agents in the absence and in the presence of a concentration 3 μmol/L of the non selective PDEs inhibitor IBMX, which effectively inhibits PDE activity in rat sinoatrial node [24]. Inhibition of PDE activity raised atrial rate by 26 ± 3 beats min^-1^ (n = 8, P<0.05), but failed to increase the chronotropic effects of either glucagon or isoproterenol in the rat right atria (Fig 6A and 6B; Table 1). To further investigate the effect of a selective PDE3 or PDE4 inhibition on the chronotropic effect of glucagon we studied the effect of this agent in the absence and the presence of each of the selective PDE3 and PDE4 inhibitors cilostamide (0.3 μmol/L) or rolipram (1μmol/L) respectively. The concentrations used, of these two inhibitors, correspond to their respective IC_50_ for PDE3 and PDE4 inhibition [9]. Rolipram increased basal atrial rate by 36 ± 6.4 beats minute^-1^ (n = 5, P<0.05) but failed to modify the effect of glucagon on atrial rate. Cilostamide neither altered the basal atrial rate nor affected the chronotropic effect of glucagon (Fig 6A, Table 1).

## Discusion

Our results demonstrate that, in the rat right atria, the transcript levels of glucagon receptors are higher in the sinoatrial node than in the working myocardium and this is associated to a positive chronotropic, but not inotropic effect of glucagon in this tissue.

Cardiostimulant effects of glucagon are considered to be due to the stimulation of glucagon receptors which, in turn, activate a Gs/adenylyl cyclase/cAMP/PKA pathway [1]. However, effects of glucagon are not uniform throughout the heart. For example, in the rat heart, glucagon increases contractility in ventricular but not in atrial myocardium [5]. Despite the lack of inotropic effect in atrial myocardium, glucagon produce a positive chronotropic effect in this tissue [7]. The reason for this discrepancy was not known but the results of the present study showing a three times higher transcript levels of glucagon receptors in sinoatrial node (the primary cardiac pacemaker), compared to the atrial myocardium, provide a plausible explanation for it. These findings were similar when we functionally identified the sinoatrial node and when the sinoatrial node was identified by the abundance of HCN levels. In contrast, the beta agonist isoproterenol increases both atrial frequency and contractility which is consistent with a same level of β-adrenoceptors which is known to exist in atrial and sinoatrial tissues [17].

The “funny current”(I*f*) is believed to play a decisive role in the pacemaker function of the sinoatrial node [10]. I*f* is an inward current carried by Na^+^ and K^+^ ions, which is specifically activated at hyperpolarized membrane potentials and regulated by cAMP [11]. The ion channels responsible for this current are HCN which are abundant in the region of the sinoatrial node but virtually absent in the rest of the atrial muscle [10]. β-Adrenoceptors stimulation, by enhancing intracellular cAMP production, activate I*f* which leads to an increase of SAN rate [11] and this effect is attenuated by I*f* inhibitors [25, 26]. Our results are consistent with this view, since the I*f* inhibitor ZD7288 reduced atrial rate, on its own, and also decreased the chronotropic effect of the β-adrenoceptor agonist isoproterenol. The fact that ZD7288 also attenuated the increase in atrial rate, induced by glucagon, indicates an involvement of I*f* in the chronotropic effect of glucagon as well. This effect is most probably due to a glucagon induced cAMP enhancement which activate HCN channels as it has been shown using recombinant HCN channels transfected by adenovirus in adult rat ventricular myocytes [27].

Sarcoplasmic reticulum Ca^2+^ release also seems to contribute to pacemaker activity in sinoatrial node by activating inward Na^+^/Ca^2+^ exchange current (NCX) which trigger the action potential and, indeed, inhibition of sarcoplasmic reticulum function with ryanodine depresses sinoatrial cells spontaneous rate [13]. This agree with our results in which the inhibitor of RyRs channels ruthenium red reduced sinoatrial node rate. However, ruthenium red failed to modify the effect of either glucagon or isoproterenol and, consequently, our data do not support the involvement of sarcoplasmic reticulum Ca^2+^ release in the chronotropic effect of these two agents.

The involvement of PKA in the chronotropic effect of either isoproterenol and glucagon has been investigated by using the two PKA inhibitors H-89 and KT-5720. H-89, but not KT-5720, reduces atrial rate which may be due to its inhibitory effect on sarcoplasmic reticulum Ca^2+^ pumps [21]. Inhibition of PKA failed to affect the chronotropic response to isoproterenol. In contrast, hindering of PKA activity with either H-89 or KT-5720 attenuated the positive chronotropic effect of glucagon. The inhibitory effect of H-89 on sarcoplasmic reticulum Ca^2+^ pumps [21] and, consequently sarcoplasmic reticulum Ca^2+^ concentrations and release, presumably do not play any role here since ruthenium red (10 μmol/L), which abolishes sarcoplasmic reticulum Ca^2+^ release [19], failed to alter the chronotropic effect of glucagon. Although both H-89 and KT-5720 are not fully selective for PKA inhibition [20, 21] and this can be considered a limitation of these experiments, the fact that both inhibitors produce similar results with glucagon and isoproterenol suggest that PKA contributes to the chronotropic effect of glucagon but not to that of isoproterenol. It is generally thought that regulation of I*f* is mediated by direct binding of cAMP to sinoatrial HCN channels, independent of phosphorylation [11], and this is in accord with our results with isoproterenol which are not altered by PKA inhibition. However, HCN channels contain PKA phosphorylation sites which indicate that PKA may also regulate them and, indeed, phosphorylation and modulation of I*f* by PKA has been reported [28, 29]. Our data with glucagon are compatible with this view. The reasons behind the different role played by PKA in the regulation of the chronotropic effects of glucagon and isoproterenol remains to be determined, but could be related to differences in cAMP distribution in sinoatrial cells microdomains, produced by each agent, which may facilitate its access to both PKA and HCN channels (in the case of glucagon) or only to HCN channels (for isoproterenol).

In addition to PKA, we also explored the possible involvement of EPAC in the chronotropic effect of glucagon and isoproterenol. EPAC, upon binding of cAMP, activates the PKC/CaMKII pathway [14]. CaMKII is involved in glucagon [30] as well as in β-adrenoceptors mediated effects [14]. It seems to play an important role in regulating basal sinoatrial node beating rate by phosphorylating RyRs and phospholamban, thereby regulating sarcoplasmic reticulum Ca^2+^ up-take and release [13]. However, in our results, inhibition of either CaMKII or PKC with KN-62 or calphostine respectively failed to modify the chronotropic effect of glucagon. Similarly, the CaMKII inhibitor KN-62 did not alter the chronotropic effect of isoproterenol. These results, together with the failure of ruthenium red to alter the effects of either glucagon or isoproterenol on sinoatrial node rate, indicate that sarcoplasmic reticulum Ca^2+^ release is not involved in the chronotropic effect of these two agents. Therefore, our results support the notion that I*f* is the predominant mechanism responsible for the positive chronotropic effect of both isoproterenol and glucagon.

Constitutive PDE activity restricts spontaneous beating rate of cardiac pacemaker cells by degrading cAMP and PDE inhibition increase I*f* by shifting the current activation to more positive potentials, an effect similar to an increase in the cAMP levels [13]. PDE are grouped into different families and, of these, PDE3 and PDE4 represent the major part of the basal PDE activity in sinoatrial node [13]. The broad spectrum PDE inhibitor IBMX and the specific PDE4 inhibitor rolipram, but not the selective PDE3 inhibitor cilostamide, increased atrial rate in our results. These results suggests that, unlike rabbit sinoatrial node cells, where PDE3 is the major constitutively active PDE in the basal state [15], in the rat it seems to be mainly regulated by PDE4. This is in agreement with previous work on cardiac contractility showing that, in rat myocardium, PDE4 is the main PDE isoenzyme regulating cAMP hydrolysis [31, 32]. It can be expected that an inhibition of PDE potentiates chronotropic responses to glucagon and isoproterenol by inhibiting hydrolysis of cAMP generated by these two agents. However, in the present work, no differences were observed between the chonotropic effects of either, glucagon or isoproterenol, in the absence and in the presence of the non selective PDE inhibitor IBMX or the selective inhibitors of either PDE3 and PDE4, cilostamide or rolipram, respectively. This is consistent with previous results showing that PDE inhibition with IBMX fails to potentiate positive chronotropic effect of the β-adrenoceptor agonists isoproterenol [33] or noradrenaline [24]. The reason why the chronotropic effects of cAMP dependent agents such as β-adrenoceptor agonists or glucagon are not affected by PDE inhibitors have not been established although it has been proposed that the cAMP increased by these agents is compartimentalized in the pacemaker cell in a microdomain different to that where PDEs are or to where PDE inhibitors used are hard to access [24].

In conclusion, our results indicate that glucagon increases rate but not contractile force in atrial myocardium and this is associated to a three times higher glucagon receptor mRNA levels in sinoatrial node than in the myocardium of the rat right atria. The chronotropic effect of glucagon seems to be due to a cAMP/PKA mediated activation of I*f* but not to sarcoplasmic reticulum Ca^2+^ release, neither it is limited by PDE activity. Glucagon is considered a cardiostimulatory agent therapeutically efficacious in the treatment of disorders characterized by cardiodepression but the involvement of the positive inotropic or chronotropic actions of glucagon for reversing cardiovascular depression is not fully known. Despite its positive inotropic effect, glucagon is not considered a useful drug for disorders characterized by a reduced cardiac output due to its lack of therapeutic effect [34, 35]. Indeed, glucagon is devoid of effect in patients with predominant impairment in myocardial contractile function, such as heart failure or circulatory shock, who responded well to other inotropic agents [36]. However, glucagon seems to be effective for treating symptomatic bradycardia induced by beta blockers or calcium channels blockers even in cases in which atropine failed to improve the patient’s condition significantly [37–39]. Atrial contractility contribute up to 30% of the cardiac output [40] and the lack of inotropic effect of glucagon on atrium reported in the present paper, as well as its relatively weak contractile effect on ventricular myocardium [5, 41, 42] may, at least partially, explain its low therapeutic efficacy as an inotropic agent. However, glucagon induced enhancement of atrial rate may effectively counteract bradicardic effect of cardiodepressant drugs. Consequently, positive chronotropic effect is, probably, the main mechanism responsible for the beneficial effects reported with glucagon in treating patients with depressed cardiac function, such as those with beta- blockers or calcium channel blocker overdose, but clinical research is demanding to confirm this view.

## Supporting Information

S1 Table Primers used in the study. Primers for GCGR were obtained from a previous publication [1].
(DOC)

S1 Fig Effect of the EPAC inhibitor ESI-09 in rat right atrial myocardium. Effect of the EPAC inhibitor ESI-09 on contractility in three (A, B and C) spontaneously beating isolated rat right atria. This agent decreases inotropy, produces tetanic contractions and completely abolishes atrial rate. These effects may be due to other action/s (yet unknown) of this agent non related to EPAC inhibition since the concentration used (0.5 μM) is below its IC_50_ for EPAC1 and EPAC2 [1], but further research is needed to clarify this point.
(DOC)
