# Supplementary material for: Glucagon Increases Beating Rate but Not Contractility in Rat Right Atrium. Comparison with Isoproterenol
Source: PLoS One. 2015 Jul 29;10(7):e0132884. doi: 10.1371/journal.pone.0132884 (PMC4519109; doi:10.1371/journal.pone.0132884)
Supplement: S1 Table — Primers for GCGR were obtained from a previous publication [1]. (DOC) [file pone.0132884.s001.doc]

| **GENE** | **FORWARD (5´-> 3´)** | **REVERSE (5´->3´)** | **LENGTH (pb)** | **REFSEC** |
| --- | --- | --- | --- | --- |
| RPLP0 (36B4) | TCGGAGGAATCCGATGAGGA | TAAGCAGGCTGACTTGGTGTG | 72 | NM_022402 |
| GCGR | TGGGATTCTGGTGGATCCTGCGT | CTTGGGCATGCTCGTCAGTCAC | 222 | XM_008768468 |
| HCN2 | GGGAATCGACTCCGAGGTCTAC | AGACTGAGGATCTTGGTGAAACG | 72 | [NM_053684.1](http://www.ncbi.nlm.nih.gov/entrez/viewer.fcgi?db=nucleotide&id=50878266) |

S1 Table

1.- Marroquí L, Batista TM, Gonzalez A, Vieira E, Rafacho A, Colleta SJ, et al. (2012) Functional and structural adaptations in the pancreatic alpha-cell and changes in glucagon signaling during protein malnutrition. Endocrinology 153: 1663-1672.
