# Supplementary material for: Glucagon Increases Beating Rate but Not Contractility in Rat Right Atrium. Comparison with Isoproterenol
Source: PLoS One. 2015 Jul 29;10(7):e0132884. doi: 10.1371/journal.pone.0132884 (PMC4519109; doi:10.1371/journal.pone.0132884)
Supplement: S1 Fig — Effect of the EPAC inhibitor ESI-09 on contractility in three (A, B and C) spontaneously beating isolated rat right atria. This agent decreases inotropy, produces tetanic contractions and completely abolishes atrial rate. These effects may be due to other action/s (yet unknown) of this agent non related to EPAC inhibition since the concentration used (0.5 μM) is below its IC50 for EPAC1 and EPAC2 [1], but further research is needed to clarify this point. (DOC) [file pone.0132884.s002.doc]

1 min

**A**


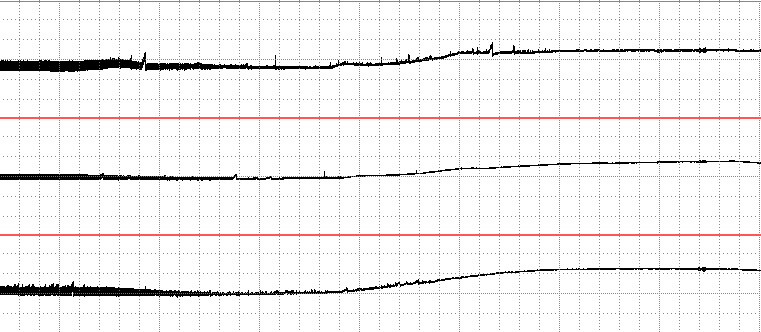


10 mN

**B**

**C**

▲

**ESI-09**

**(0.5µM)**

S1 Fig.:

1.- Almahariq M, Tsalkova T, Mei FC, Chen H, Zhou J, [Sastry SK](http://www.ncbi.nlm.nih.gov/pubmed/?term=Sastry SK%5BAuthor%5D&cauthor=true&cauthor_uid=23066090), et al. (2013) A novel EPAC-specific inhibitor suppresses pancreatic cancer cell migration and invasion. Mol Pharmacol 83: 122-128.
